# Enhancing Self-Care Confidence Among Colorectal Cancer Patients: Insights From Postoperative Experiences

**DOI:** 10.7759/cureus.95525

**Published:** 2025-10-27

**Authors:** Jimena Alvarez del Castillo Gonzalez, Saakshi Bansal, Lara Nassar, Nico-Karlo Plete, Shiela Joy Bellen, Victoria Nelson, Nathan Carter, Harriet Hargreaves, Shengyang Qiu, Shahnawaz Rasheed

**Affiliations:** 1 Colorectal Surgery, The Royal Marsden Hospital, London, GBR; 2 Urology, Lister Hospital, Stevenage, GBR; 3 Pain Medicine, The Royal Marsden Hospital, London, GBR; 4 Dietetics, The Royal Marsden Hospital, London, GBR; 5 Stoma Care, The Royal Marsden Hospital, London, GBR

**Keywords:** after surgery recovery, patient education, post-colorectal cancer surgery experience, post-surgery leaflet, quality improvement project

## Abstract

Background: Enabling patients to participate more actively in their health through better access to information is central to good medical practice. This project seeks to bridge existing gaps in patient knowledge regarding postoperative care after colorectal cancer surgery by introducing strategies aimed at enhancing the overall recovery experience.

Materials and methods: We conducted an electronic survey to gather feedback from patients who were surgically managed for colorectal cancer at a single centre between January and September 2023. Using a scale of 1-10 (with 10 being the best score), we enquired about their experience with the education they received in the following five areas: pain management, stoma care, wound management, dietary advice, and contacting their surgical team. We also asked about their preferences regarding the form of delivery for post-discharge information. Our multidisciplinary team developed a physical leaflet delivered alongside a face-to-face discussion. This intervention was implemented for patients undergoing surgery between March and May 2024, and two post-intervention surveys were conducted to evaluate their experience: one day before discharge and two weeks later.

Results: A total of 33 feedback forms were obtained prior to the intervention, while 15 forms were collected from the post-intervention group. Overall, most participants rated their experience above a score of seven out of 10 in all five domains before the intervention (BI) and above a nine out of 10 after the intervention (AI). The calculated mean value for stoma care was 8.73 ±0.712 (BI, confidence interval (CI) 95%, n=19) vs. 9.33 ±0.354 (AI, CI 95%, n=15); pain management was 8.72 ±0.608 (BI, CI 95%, n=31) vs. 9.27 ±0.391 (AI, CI 95%, n=15); wound management was 8.06 ±0.836 (BI, CI 95%, n=30) vs. 9.33 ±0.354 (AI, CI 95%, n=15); dietary advice was 7.20 ±1.018 (BI, CI 95%, n=29) vs. 9.20 ±0.331 (AI, CI 95%, n=15); and contacting their surgical team was 8.25 ±0.709 (BI, CI 95%, n=32) vs. 9.40 ±0.331 (AI, CI 95%, n=15). Data are represented as absolute percentages and mean ± SEM (standard error of the mean). When asked whether receiving psychological support during their cancer journey would be beneficial, before the intervention group, 61.3% (n=19) of the 31 respondents answered positively. Within this group, the majority of patients (n=28, 87.5%) expressed a preference for face-to-face advice following discharge, with fewer opting for electronic instructions (n=14, 43.8%), leaflets (n=13, 40.6%), phone advice (n=6, 18.8%), or online videos (n=5, 15.6%). Following the intervention, all 15 respondents in the final survey reported that the leaflet helped address most of their concerns prior to discharge. They also agreed that it covered key aspects of their post-surgical recovery and enhanced their overall recovery experience.

Conclusion: Effective communication is a key aspect in the healthcare environment for reducing stress and improving understanding. By offering tailored educational support, clinicians can help colorectal cancer patients feel more confident, supported, and autonomous during recovery.

## Introduction

Colorectal cancer is the third most common cancer globally and the second leading cause of cancer-related deaths, according to the World Health Organisation [1]. Due to its clinical presentation and anatomical location, the management of colorectal cancer often involves complex surgical procedures, which can lead to a challenging postoperative recovery [2]. This recovery phase typically requires the involvement of a highly specialised multidisciplinary team responsible for managing pain, providing tailored nutritional support, offering wound and stoma care, and giving psychological support [3]. Although healthcare providers play a leading role in the postoperative pathway, involving patients in decision-making and their recovery plans can significantly improve the quality of care, increase satisfaction with postoperative outcomes, and help reduce unnecessary hospital costs [4,5].

During hospital stays, factors such as fatigue, slow return to normal eating, the body's physical response to surgery, and unfamiliarity with medical information can affect a patient's ability to fully understand and follow clinical advice [6,7]. Therefore, discharge planning should be comprehensive and person-centred [8]. It should aim to present key information in a clear and structured manner, especially for those without a clinical background, particularly during the first days following discharge to a non-hospital setting [9,10]. Additionally, ensuring patients have easy access to contact their healthcare team is a crucial component of the recovery pathway [11]. In interviews conducted six weeks after surgery, patients frequently identified contacting the surgical team as their main concern in the early post-discharge period [6].

Patients have expressed the importance of having clear information about symptom management, medication side effects, contact details of the surgical team, and available psychological support during their recovery [12,13]. Previous research has highlighted ongoing gaps in the provision of post-discharge information for colorectal cancer patients [6], underscoring the need for clearer guidance on what constitutes a typical recovery, when and how to seek help, and how to effectively deliver this information [14].

This project aimed to improve patients' experiences and confidence in managing their post-surgical recovery by implementing diverse and targeted strategies for sharing relevant knowledge. The project specifically sought to address existing gaps in patient education and support throughout their recovery journey.

## Materials and methods

This project followed a five-step process. The first step included gathering feedback from patients with a history of colorectal surgery to understand their overall postoperative experience. The next step involved collaborating with various healthcare teams involved in post-colorectal surgery care to design a leaflet aimed at addressing common challenges faced after hospital discharge. A pilot group of patients was then selected to review and evaluate the intervention that had been created. Based on their feedback, suggestions were addressed, and additional resources were incorporated to improve the content. Finally, the revised leaflet was explained to all patients who underwent colorectal surgery.

The participants selected for the initial post-surgery survey had undergone one of the various colorectal cancer surgeries performed at a single cancer centre in the United Kingdom between January and September 2023. These procedures included open or robotic total pelvic exenteration (TPE), robotic or open extra-levator abdominoperineal resection (ELAPE), total mesorectal excision (TME), hemi-, subtotal, or total colectomy, and robotic or open anterior or low rectal resections. Patients who underwent open or laparoscopic ileostomy/colostomy formation or reversal were also included.

In contrast, those who had undergone only an examination under anaesthesia (EUA) or colonoscopy were excluded, as these procedures typically do not involve a complex recovery pathway.

The survey content was thoroughly reviewed by consultant colorectal surgeons and senior surgical registrars and further assessed by the hospital's Clinical Audit and Quality Improvement Committee (CAQIC) (see Appendix 1 for the first survey template). After receiving approval for distribution, the survey was sent to patients via email. The electronic responses collected during the first week of October 2023 were analysed to assess patients' experiences with the information provided upon discharge, specifically by the colorectal team, dietitians, pain specialists, and stoma nurses. The survey also explored the need for psychological support and identified preferred strategies for delivering post-discharge information.

In the following phase, carried out from October to December 2023, collaboration took place with multidisciplinary specialist teams, including colorectal surgeons, dietitians, stoma care nurses, the pain team, and psychological support services. A designated specialist from each team developed content relevant to their area of expertise under the supervision of their departmental lead. The resulting comprehensive leaflet included information on each team's role in the patient's recovery, common symptoms within their area, general guidance on managing those symptoms, available support services, and contact details for further assistance.

To evaluate its impact, a pilot trial was conducted from March to May 2024, during which the leaflet was provided to patients who had recently undergone colorectal surgery along with a feedback questionnaire (see Appendix 2 for the first post-intervention survey template). This was followed by a second round of data collection using a multiple-choice feedback survey (see Appendix 3 for the final survey template). Based on their observations, the leaflet was refined and improved. The final version received formal approval from the colorectal consultants and the CAQIC for implementation across the hospital.

The relationships between results before and after the intervention were evaluated using mean values ±SEM and a 95% CI. Analyses were performed using IBM SPSS Statistics for Windows, Version 28 (Released 2021; IBM Corp., Armonk, New York).

## Results

A total of 92 patients underwent colorectal cancer surgery at a highly specialised hospital in London, United Kingdom between January and September 2023. From this cohort, 33 electronic feedback forms were collected in October 2023, generally reflecting a positive experience with the information provided by the various teams involved in their discharge process. A descriptive analysis was conducted to comprehensively examine the data.

**Figure 1:**
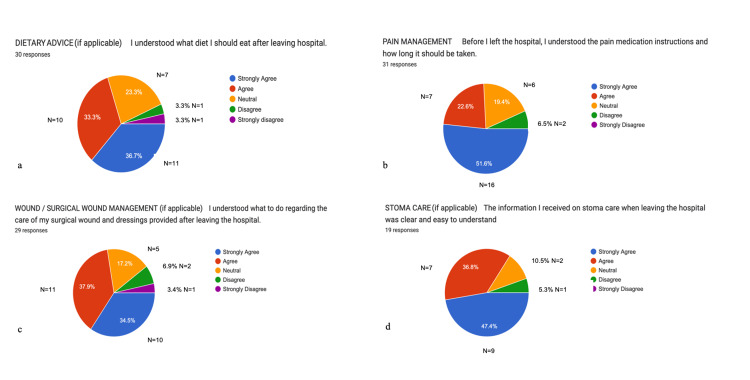
Data collected from pre-intervention feedback forms Data provided by electronic pre-intervention feedback forms. A total of 119 post-surgical feedback responses are represented in a pie chart from 33 survey emails collected (Figures a-d). Values are shown in percentages (%). Charts (a-d) show the patients' understanding of the information provided prior to discharge from the hospital regarding dietary advice in 70% (n=30), pain management 73.6% (n=31), wound management 72.4% (n=29), and stoma care 78.2% (n=19); 73.55 ±2.922 (CI=95%, n=109) on average (mean values) agreed to understand the information provided on the area mentioned. Data are represented as absolute percentages and mean±SEM. CI = confidence interval, SEM = standard error of the mean.

On average, 75.2% (n=109) of responses indicated that participants felt confident with the information received regarding pain control, stoma care, wound management, and dietary guidance after discharge. Results are summarised in Figure 1 below.

**Figure 2:**
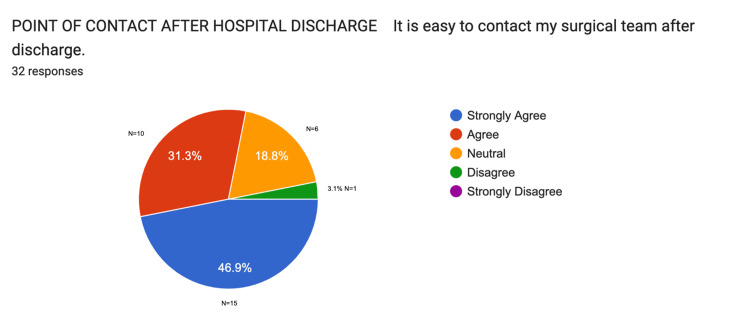
Patient access to surgical team support pre-intervention Data provided by electronic feedback forms pre-intervention. A total of 32 post-surgical feedback responses are represented in a pie chart, based on 33 survey emails collected. Values are shown in percentages (%). The chart illustrates how easily they were able to reach their surgical team after surgery.

As part of the post-discharge follow-up, participants were asked how easy it was to contact their surgical team after leaving the hospital. A total of 78.2% of participants, based on mean values (n=32), reported that they were able to communicate with their team easily once at home. Results are represented in Figure 2.

**Figure 3:**
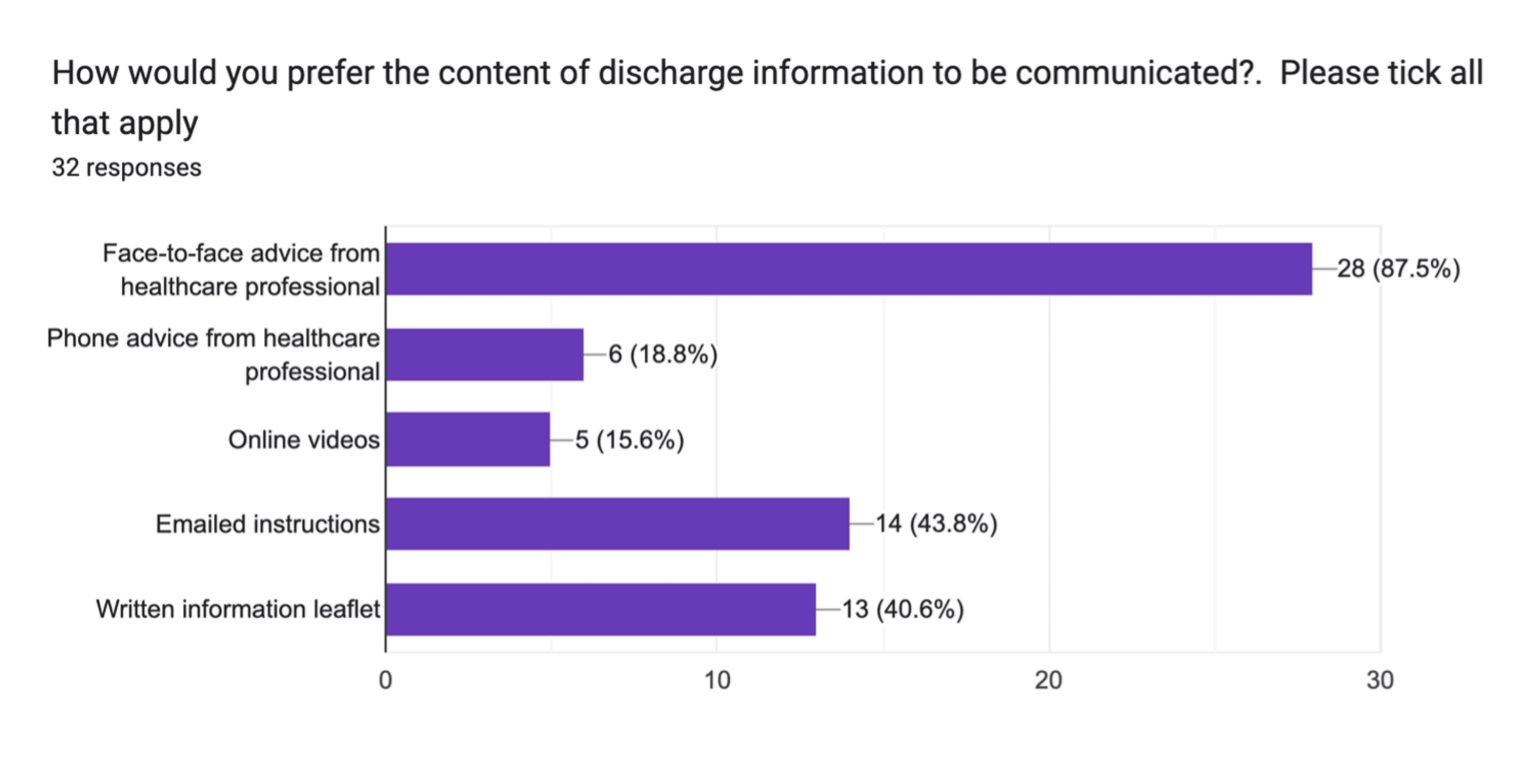
Discharge information: patient communication preferences pre-intervention Data provided by electronic feedback forms pre-intervention. A total of 32 post-surgical feedback responses are represented in a graph from 33 survey emails collected. Values are shown in percentages (%). The chart illustrates the preferred method for delivering the discharge information pack.

Participants were asked about their preferred methods for receiving postoperative information. The majority (87%, n=32) favoured face-to-face advice from a healthcare professional. This was followed by 44% (n=14) who preferred receiving instructions via email and 41% (n=13) who selected written materials. Phone advice and online videos were the least commonly preferred options, with 19% (n=6) and 15% (n=5) of respondents choosing these options, respectively. These preferences are illustrated in Figure 3.

**Figure 4:**
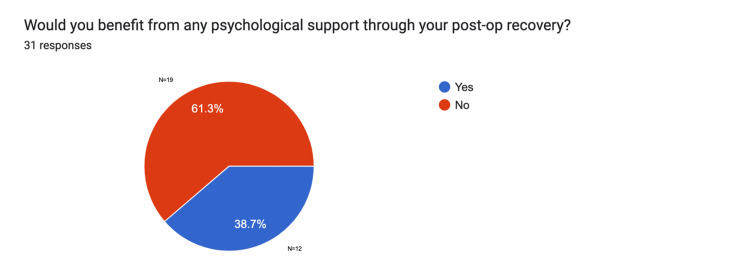
Patient preferences for psychological support pre-intervention Data provided by electronic feedback forms pre-intervention. A total of 31 post-surgical feedback responses are represented in a pie chart, based on 33 survey emails collected. Values are shown in percentages (%). The chart indicates whether patients would benefit from psychological advice during their postoperative process.

As part of the questionnaire, participants were asked whether they believed psychological support would be beneficial during their postoperative recovery. Only 36.7% (n=12) of respondents agreed with this option. The results are illustrated in Figure 4.

In the final question, we explored the overall patient experience regarding the information provided by the different multidisciplinary teams at the time of discharge.

**Figure 5:**
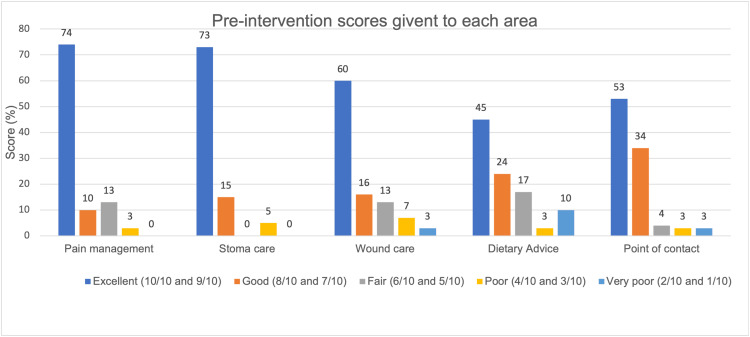
Scores given by patients prior to implementation of the intervention Data have been represented in a bar chart created from electronic feedback forms, with the percentage (%) of respondents pre-intervention reflecting the score given to their experience in each area (dietary advice, wound care, pain management, stoma care, and point of contact). A total of 141 responses were collected. The bar chart indicates that, overall, most participants rated their experience above a score of 7 in all five domains prior to the intervention. The mean value for pain management was 8.72 ±0.608 (CI 95%, n=31); stoma care 8.73 ±0.712 (CI 95%, n=19); wound management 8.06 ±0.836 (CI 95%, n=30); dietary advice 7.20 ±1.018 (CI 95%, n=29); and contacting their surgical team 8.25 ±0.709 (CI 95%, n=32). Data are represented as absolute percentages and mean ± SEM. CI = confidence interval, SEM = standard error of the mean.

This question included a quantitative component: "On a scale of 1 to 10, how would you rate your experience in the following areas?" covering dietary advice, wound management, pain management, stoma care, and point of contact. On average (based on mean values), 82.26% (n=141) of responses scored above 7 out of 10 concerning the information and support provided across these five domains of postoperative care. The results are presented in Figure 5.

Assessing the clarity, organisation, and content of the leaflet created before leaving the hospital

For the second part of the project, feedback was gathered from 15 postoperative patients between March and April 2024, one day prior to their hospital discharge. A survey was designed to assess the clarity and organisation of the leaflet's content, as well as whether it addressed most of their recovery-related concerns before leaving the hospital.

**Figure 6:**
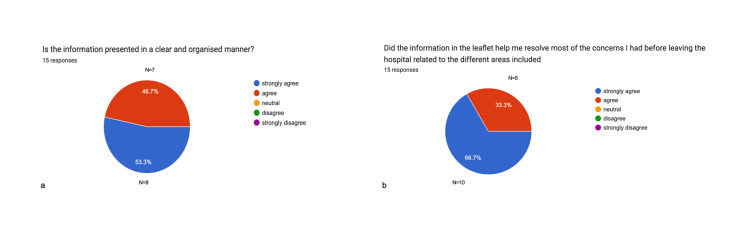
Leaflet organisation and its effectiveness in addressing patient concerns post-intervention Data have been represented in pie charts (a-b) from feedback forms after the first leaflet was given, showing values in %. A total of 30 responses were collected. Both charts (a-b) show 100% of patients (n = 15) agree that the leaflet provided after discharge was organised in a clear form, and this resolved most of their clinical concerns before leaving the hospital.

Overall, 100% (n=15) of participants agreed that the information presented in the leaflet was clear and well-organised and that it helped resolve most of their concerns before discharge. The results are presented in Figure 6.

Two-week follow-up assessment of patient experience after the intervention

For the final phase of the project, feedback was obtained from the same 15 patients during their follow-up clinic appointment, which took place two weeks after hospital discharge.

**Figure 7:**
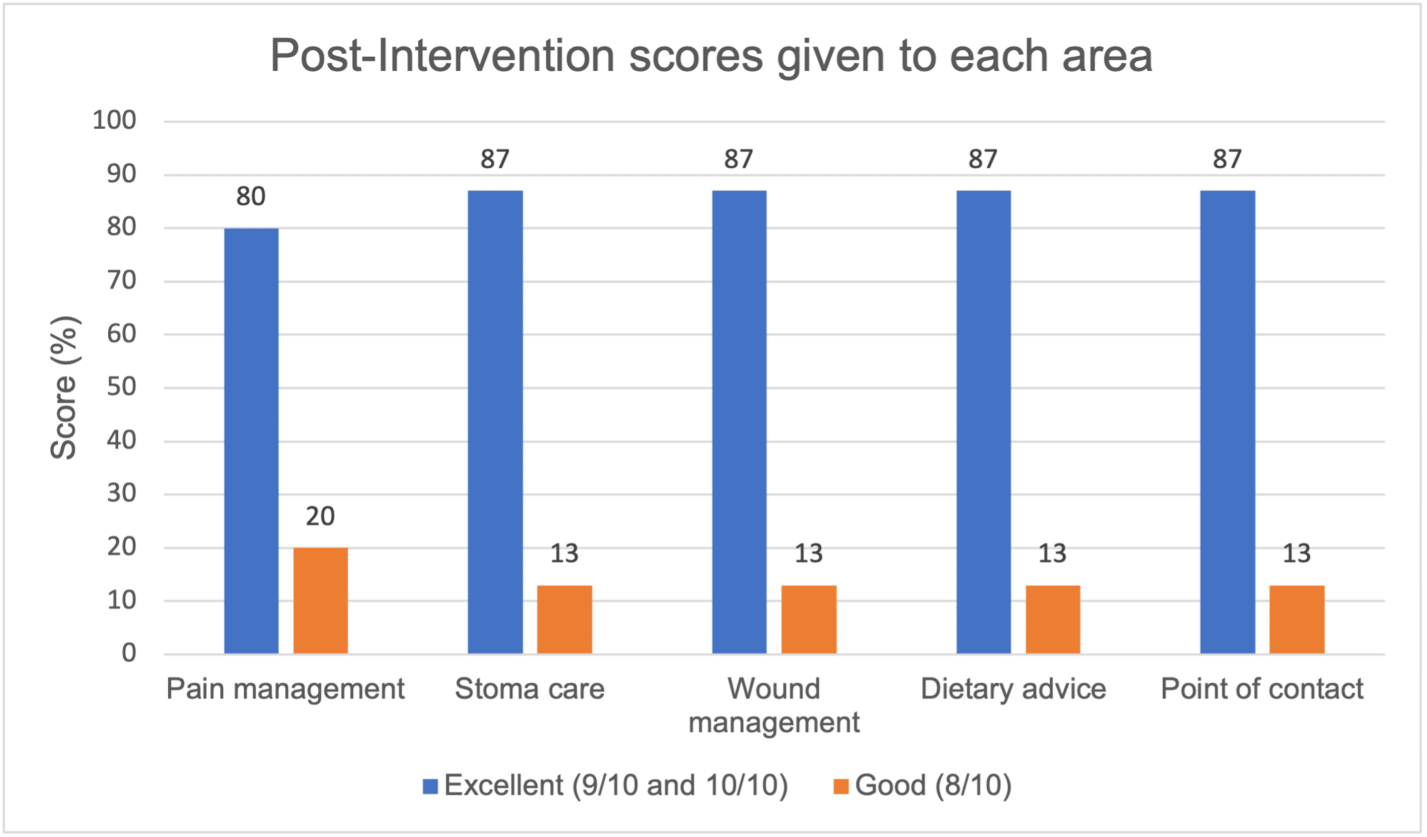
Post-intervention experience scores given to each area assessed Data have been represented in a bar chart created from electronic feedback forms from 15 respondents, reflecting the scores given to their experience in each area (dietary advice, wound care, pain management, stoma care, and point of contact). The bar chart shows that, overall, most participants rated their experience above a score of 9 in all five domains before the intervention. The mean value for pain management was 9.27 ± 0.391 (CI 95%, n=15); stoma care 9.33 ± 0.354 (CI 95%, n=15); wound management 9.33 ± 0.354 (CI 95%, n=15); dietary advice 9.20 ± 0.331 (CI 95%, n=15); and contacting their surgical team 9.40 ± 0.331 (CI 95%, n=15). Data are represented as absolute percentages and means ± SEM. CI = confidence interval, SEM = standard error of the mean.

**Figure 8:**
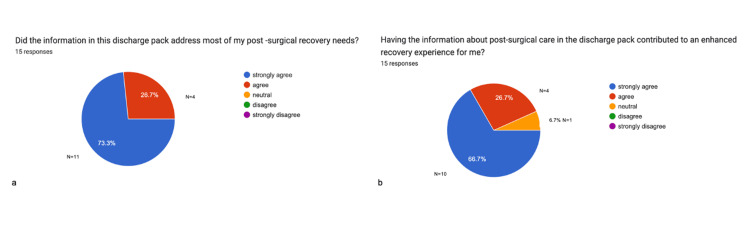
Impact of discharge information on post-surgical recovery experience after intervention Data provided by feedback forms after intervention. A total of 15 post-surgical feedback responses are represented in a pie chart (a-b). Values are shown in percentages (%). The chart represents whether the information in the discharge pack addresses most of their post-surgical recovery needs and if this contributed to an enhanced recovery experience.

**Figure 9:**
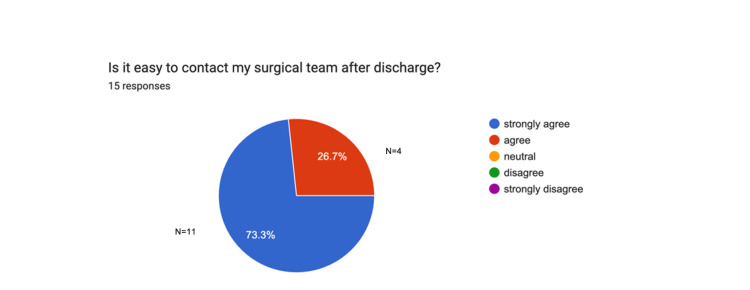
Patient access to the surgical team post-discharge after intervention Data provided by feedback forms after intervention. A total of 15 post-surgical feedback responses are represented in a pie chart. Values are shown in percentages (%). The chart illustrates the ease of contacting the surgical team after discharge.

Following the intervention, 100% (n=15) of participants reported an improved experience across all five key areas: pain management, stoma care, wound management, dietary advice, and point of contact after discharge, with each area rated above 8 out of 10 (results illustrated in Figure 7). Additionally, all patients (100%, n=15) agreed that the leaflet addressed most of their post-surgical recovery needs, and 93.4% (n=15) stated that this material contributed to an enhanced recovery process (responses are represented in Figure 8). Finally, all participants reported that contacting their surgical team post-discharge was easier after receiving the intervention (see responses in Figure 9).

## Discussion

After major colorectal surgery, several key areas of the recovery phase must be carefully managed to ensure optimal postoperative outcomes. As recovery continues beyond hospital discharge, patients often face unexpected challenges or uncertainties. These may arise from a lack of clinical background or from the anxiety and stress associated with navigating an unfamiliar situation [12-15]. To support patients during this period, it is essential that they understand how to care for themselves, recognise warning signs, and know when and how to contact healthcare providers. Patient education has consistently proven to be a valuable and accessible tool in this regard [16].

In this project, patients reported a reasonable level of confidence in the information provided by various healthcare teams regarding five crucial aspects of post-surgical care. These areas included the diet they should follow after surgery, pain medications needed to be taken to manage symptoms, their stoma care, how to look after their surgical wound in the following days after surgery, and how to reach psychological support if needed [17]. While initial feedback was generally positive, the project revealed opportunities to further enhance patient understanding and confidence in these areas.

As part of our strategy to improve information delivery, patients highlighted a preference for receiving information face-to-face, supported by written materials such as emails or leaflets. This reinforces the vital role that healthcare providers play in education and highlights the effectiveness of providing structured, accessible written resources [18]. As a result, the leaflet was developed collaboratively by five specialist teams: pain management, stoma care, dietetics, psychology, and the colorectal surgical team. We recognised that medical jargon and poor structuring can lead to confusion and disengagement. All participants agreed that the leaflet was clear, well-organised, and effectively addressed their concerns prior to discharge. This clarity likely contributed to the improved patient experience observed following the intervention.

Another important area assessed was patients’ ability to contact their surgical team after discharge. This proved to be a key factor in reducing uncertainty and promoting confidence during recovery. Although we did not explore the specific reasons patients sought contact, feedback post-intervention suggested a noticeable improvement in communication [19]. We believe this may be due to increased awareness of what to expect and how to respond to emerging concerns, supported by having clear contact information included in the discharge pack. Future studies could examine the nature of these interactions to better understand how they relate to recovery outcomes.

The overall aim of this project was to assess whether a structured discharge pack could improve the postoperative experience for colorectal cancer patients. The results showed that the intervention, which included a leaflet, increased patients’ understanding of the recovery process, addressed most of their concerns, and enhanced their overall experience, including ease of communication with the surgical team.

Limitations

Some limitations were identified during the data collection process, which affected the sample size. The initial questionnaire included only patients who had undergone major colorectal surgery, excluding those who had day-case procedures such as colonoscopy or EUA. This reduced the number of eligible participants.

Additionally, the first round of feedback included patients who had surgery up to nine months prior to the survey. This time gap may have affected their ability to recall specific aspects of their recovery. We attempted to mitigate this by designing clear and focused questions.

We recognise physiotherapy as an important element of recovery after surgery. This section was not included in the final leaflet due to the diversity of surgical procedures used in the management of colorectal cancer, where recommendations need to be individualised for each patient.

## Conclusions

Overall, the recovery period following major colorectal surgery presents unique challenges, particularly once patients return home and no longer have immediate clinical support.

This project demonstrated that providing a structured, easy-to-understand discharge pack, combined with a face-to-face explanation, can significantly enhance the patient experience. By improving knowledge, addressing common concerns, and facilitating communication with healthcare teams, such interventions can enhance patients' overall recovery experience after major colorectal surgery.

## Appendices

Appendix 1

Pre-intervention patient questionnaire 

Please state to what extent you agree or disagree with these statements about your discharge information. 

DIETARY ADVICE (if applicable)   

Q1. I understood what diet I should eat after leaving the hospital.  

Strongly Agree 5

Agree 4

Neutral 3

Disagree 2

Strongly Disagree 1

Q2. The information I received on diet when leaving the hospital was clear and easy to understand.  

Strongly Agree 5

Agree 4

Neutral 3

Disagree 2

Strongly Disagree 1  

WOUND / SURGICAL WOUND MANAGEMENT (if applicable)  

Q3. I understood what to do regarding the care of my surgical wound and dressings provided after leaving the hospital.  

Strongly Agree 5

Agree 4

Neutral 3

Disagree 2

Strongly Disagree 1

PAIN MANAGEMENT     

Q4. Before I left the hospital, I understood the pain medication instructions and how long it should be taken.  

Strongly Agree 5

Agree 4

Neutral 3

Disagree 2

Strongly Disagree 1

STOMA CARE (if applicable)  

Q5. The information I received on stoma care when leaving the hospital was clear and easy to understand

Strongly Agree 5

Agree 4

Neutral 3

Disagree 2

Strongly Disagree 1

POINT OF CONTACT AFTER HOSPITAL DISCHARGE  

Q6. It is easy to contact my surgical team after discharge.  

Strongly Agree 5

Agree 4

Neutral 3

Disagree 2

Strongly Disagree 1

Q7. How would you prefer the content of discharge information to be communicated?.   

Face-to-face advice from healthcare professional  

Phone advice from healthcare professional  

Online videos

Emailed instructions  

Written information leaflet  

Q8. Would you benefit from any psychological support through your post-op recovery?  

Yes or No

Q9. On a scale of 1-10 (1 = very poor experience, 10 = very good experience), how would you rate the following areas?

Dietary Advice

Wound management

Pain management

Stoma care

Point of contact

Appendix 2

Post-intervention in-patient questionnaire (before hospital discharge)

Please state to what extent you agree or disagree with these statements about your discharge information. 

Q1. Is the information presented in a clear and organised manner?

Strongly Agree 5

Agree 4

Neutral 3

Disagree 2

Strongly Disagree 1

Q2. Did the information in the leaflet help me resolve most of the concerns I had before leaving the hospital related to the different areas included?

Strongly Agree 5

Agree 4

Neutral 3

Disagree 2

Strongly Disagree 1

Appendix 3

Post-intervention outpatient questionnaire (2 weeks after hospital discharge)

Please state to what extent you agree or disagree with these statements.   

Q1. Did the information in this leaflet address most of my post-surgical recovery needs?

Strongly Agree 5

Agree 4

Neutral 3

Disagree 2

Strongly Disagree 1

Q2. Having the information about post-surgical care in the leaflet contributed to an enhanced recovery experience for me.

Strongly Agree 5

Agree 4

Neutral 3

Disagree 2

Strongly Disagree 1

Q3. Is it easy to contact my surgical team after discharge?  

Strongly Agree 5

Agree 4

Neutral 3

Disagree 2

Strongly Disagree 1

Q4. On a scale of 1-10 (1 = very poor experience, 10 = very good experience), what was your experience of the following areas?

Dietary Advice

Wound management

Pain management

Stoma care

Point of contact
